# TCF7L1 Modulates Colorectal Cancer Growth by Inhibiting Expression of the Tumor-Suppressor Gene EPHB3

**DOI:** 10.1038/srep28299

**Published:** 2016-06-23

**Authors:** Matthew Murphy, Sujash S. Chatterjee, Sidharth Jain, Manpreet Katari, Ramanuj DasGupta

**Affiliations:** 1 New York University Langone Medical Center, Perlmutter Cancer Center, Department of Stem Cell Biology, 522 1st Ave., Smilow Research Building, Rm 1206 New York, NY, 10016, USA; 2 University of Houston, Department of Biochemistry and Biology, Science & Research Building 2, 3455 Cullen Blvd #342, Houston, TX 77004, USA; 3 New York University, Department of Biology, 1009 Silver Center, 100 Washington Square East, New York, NY, 10003, USA; 4 Genome Institute of Singapore, Cancer Therapeutics and Stratified Oncology, 60 Biopolis Street, #02-01, Genome, 138672, Singapore

## Abstract

Dysregulation of the Wnt pathway leading to accumulation of β-catenin (CTNNB1) is a hallmark of colorectal cancer (CRC). Nuclear CTNNB1 acts as a transcriptional coactivator with TCF/LEF transcription factors, promoting expression of a broad set of target genes, some of which promote tumor growth. However, it remains poorly understood how CTNNB1 interacts with different transcription factors in different contexts to promote different outcomes. While some CTNNB1 target genes are oncogenic, others regulate differentiation. Here, we found that TCF7L1, a Wnt pathway repressor, buffers CTNNB1/TCF target gene expression to promote CRC growth. Loss of TCF7L1 impaired growth and colony formation of HCT116 CRC cells and reduced tumor growth in a mouse xenograft model. We identified a group of CTNNB1/TCF target genes that are activated in the absence of TCF7L1, including EPHB3, a marker of Paneth cell differentiation that has also been implicated as a tumor suppressor in CRC. Knockdown of EPHB3 partially restores growth and normal cell cycle progression of TCF7L1-Null cells. These findings suggest that while CTNNB1 accumulation is critical for CRC progression, activation of specific Wnt target genes in certain contexts may in fact inhibit tumor growth.

The Wnt/CTNNB1 pathway is highly conserved and regulates a wide range of cellular and developmental processes^1^,^2^. In mammals, one of the key functions of this pathway is to regulate homeostasis of the intestine and colon, where rapid cellular turnover is required to replace epithelial cells subject to the harsh conditions of the intestinal tract^3^.

In normal cells, levels of CTNNB1 protein are tightly regulated by a “destruction complex” that phosphorylates CTNNB1 at key residues, targeting it for ubiquitination and subsequent proteasomal degradation^4^. Binding of extracellular Wnt proteins to Frizzled and LRP receptors activates a signal transduction cascade that inhibits the destruction complex, allowing cytosolic CTNNB1 protein to accumulate. Stabilized CTNNB1 translocates to the nucleus, where it acts as a transcriptional coactivator. Apart from its function in the nucleus, CTNNB1 also localizes to the membrane, where it links E-cadherin to α-catenin at adherens junctions.

Over 90% of colorectal tumors contain mutations that disrupt this regulation and cause aberrant accumulation of CTNNB1 protein^5^. Most of these mutations compromise the function of APC (adenomatous polyposis coli), a member of the destruction complex that was identified as a driver of a familial CRC^6^, but mutations are also frequent in CTNNB1 phosphorylation sites that prevent its degradation. This Wnt pathway activation is thought to be the first step in tumorigenesis for a majority of CRC patients^7^.

Nuclear CTNNB1 interacts with a variety of proteins; the best characterized are the T cell factor/lymphoid enhancer factor (TCF/LEF) transcription factors. This family contains four members, each of which recognize and bind to a shared DNA motif (“Wnt-responsive elements,” or WREs) through an HMG-box domain^8^, and interact with the TLE/Groucho family of transcriptional repressors^9^. Of the four factors, TCF7L1 (also known as TCF-3) has the strongest binding to TLE proteins, making it the strongest repressor of the family^10^.

The Wnt pathway has been viewed as an attractive clinical target, as it is frequently activated in a number of cancer types, particularly colorectal cancer^11^,^12^. This view is supported by findings that expression of oncogenes such as MYC and CCND1, which promote cell growth and cell cycle progression, is activated by the Wnt pathway via CTNNB1/TCF^13^,^14^. However, evidence has shown that the effects of Wnt/CTNNB1 signaling are context-specific, and it can either promote or inhibit cancer progression^15^.

This is illustrated by recent literature showing that activation of the Wnt pathway via TCF7L1 knockdown slowed growth of breast cancer xenograft tumors^16^ and reduced proliferation of acute lymphoblastic leukemia cells^17^. In addition, a number of genes which have been identified as direct targets of CTNNB1/TCF transcriptional complexes correlate with improved CRC patient survival and have been shown to reduce CRC cell and tumor growth, such as CDX2^18^ and EPHB3^19^,^20^.

In light of these observations, we wanted to investigate the specific function of TCF7L1 in colorectal cancer models to determine how it regulates cell proliferation, tumor growth, and CTNNB1/TCF target gene expression.

## Results

### TCF7L1 is a nuclear repressor of Wnt signaling in colorectal cancer cells

A vast majority of colorectal cancer (CRC) tumors contain mutations in genes encoding members of the Wnt signaling pathway, and nearly all of these mutations lead to stabilization and excessive accumulation of CTNNB1 protein. HCT116 cells, isolated from colorectal carcinoma, are heterozygous for an activating mutation in CTNNB1 (ΔS45) that prevents it from being phosphorylated by casein kinase 1 alpha (CSNK1A1), which normally marks the protein for further phosphorylation and proteasomal degradation^4^.

**Figure 1:**
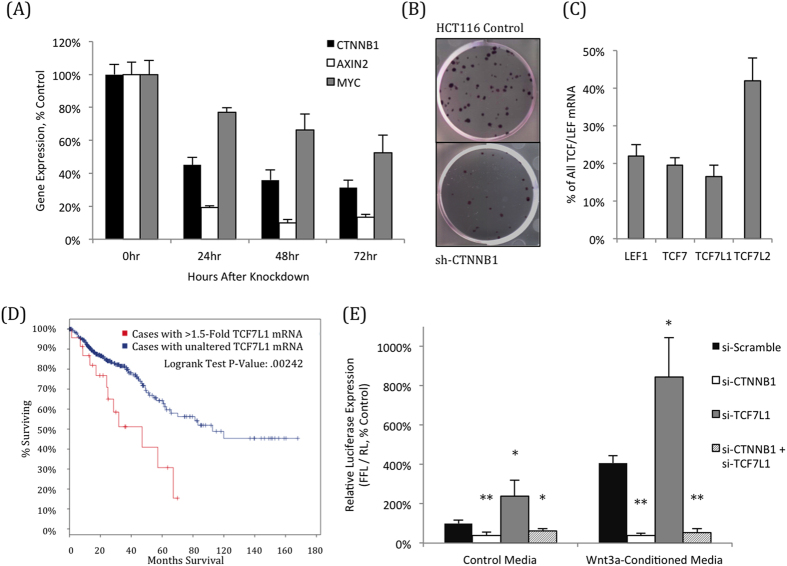
TCF7L1 represses Wnt-dependent transcription in HCT116 cells. (**A**) Knockdown of CTNNB1 reduces expression of AXIN2 and MYC, two Wnt pathway target genes that are direct targets of CTNNB1/TCF. (**B**) Knockdown of CTNNB1 causes a massive reduction in HCT116 growth and colony formation. (**C**) HCT116 cells express all four TCF/LEF transcription factors, shown by qPCR as a percentage of the total combined TCF/LEF mRNA. (**D**) CRC patients whose tumors had elevated TCF7L1 mRNA levels (at least 1.5-fold above average) had a significantly shorter survival (47 months) than those with normal TCF7L1 expression (93 months). Patient data utilized was from the TCGA provisional colorectal adenocarcinoma database, accessed via cbioportal.org. (**E**) Knockdown CTNNB1 reduces expression TOPFlash, a Wnt pathway transcriptional reporter. Conversely, knockdown of TCF7L1 activates reporter expression, confirming its role as a transcriptional repressor in HCT116 cells, even in the presence of exogenous Wnt3a. Activation by TCF7L1 knockdown and Wnt3a addition were both dependent on CTNNB1 expression. (*P < 0.05, **P < 0.01, compared to si-Scramble for the respective media condition).

HCT116 cells require CTNNB1 for normal growth, as its knockdown leads to rapid reduction in Wnt pathway target genes AXIN2 and MYC (Fig. 1A Supplementary Fig. S1), along with a reduction in cell growth and colony formation (Fig. 1B). While CTNNB1 is critical both for CRC disease progression and cell growth, the role of individual TCF/LEF family members remains unclear^8^. HCT116 cells expresses each of the four TCF/LEF transcription factors (Fig. 1C), and are responsive to transfection and transduction, making it an ideal cell line to use to query the function of these individual factors.

Much of the research on downstream Wnt signaling focuses on the role of “activating” TCF/LEF factors, which promote transcription with CTNNB1^8^. However, we were curious to determine the importance of TCF7L1, a known transcriptional repressor of the Wnt pathway, particularly because patients with elevated TCF7L1 expression have poorer clinical outcomes, with a median survival of just 47 months, compared to 93 months in patients with unaltered expression (Fig. 1D). Of the four TCF/LEF family members, TCF7L1 has the strongest affinity for the TLE/Groucho family of co-repressors^10^ and competes with other CTNNB1/TCF complexes for binding to TCF target genes^21^.

In mouse embryonic stem cells and breast cancer cells, it has been shown that CTNNB1 can regulate TCF7L1 protein levels by exporting it from the nucleus to the cytoplasm, where it is degraded^22^. However, in HCT116 colorectal cancer cells, knockdown of CTNNB1 did not increase TCF7L1 protein levels, and CTNNB1 stabilization by addition of Wnt3a showed only a modest decrease in TCF7L1 protein (Supplementary Fig. S1).

As expected, TCF7L1 acts as a transcriptional repressor in CRCs; its knockdown resulted in a significant increase in the STF14 Wnt reporter activity that was dependent on the presence of CTNNB1, both in control media and in the presence of exogenous Wnt3a (Fig. 1E). Importantly, TCF7L1 protein was localized to the nucleus of HCT116 cells, even in the presence of exogenous Wnt3a, which explains its ability to act as a Wnt pathway repressor despite high levels of stable CTNNB1 protein (Supplementary Fig. S1).

While Wnt3a addition further stabilized CTNNB1 protein and stimulated Wnt pathway activity, this did not cause significant changes in HCT116 cell growth or colony formation (Supplementary Fig. S1).

### Loss of TCF7L1 reduces growth of colorectal cancer cells

**Figure 2:**
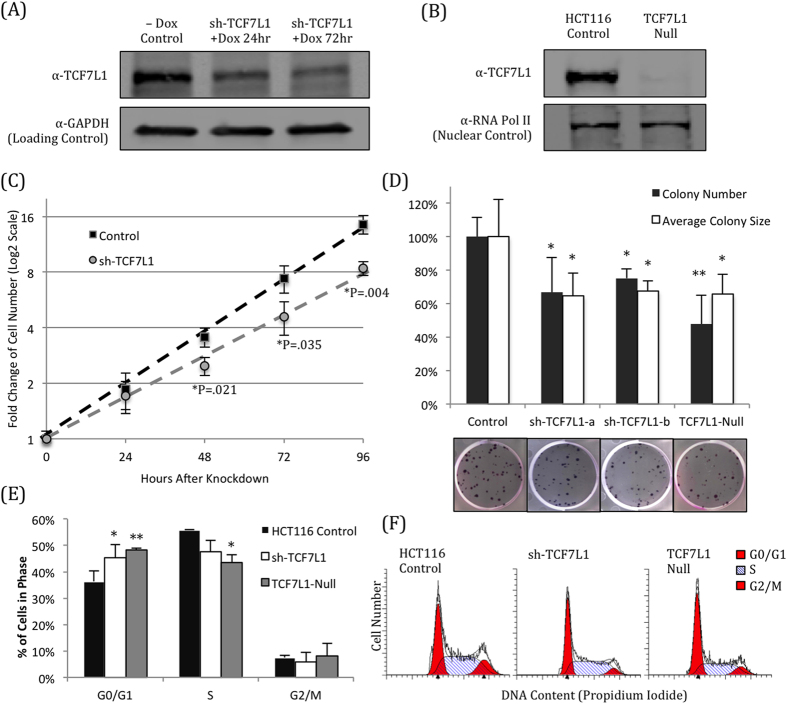
Loss of TCF7L1 reduces HCT116 cell growth and proliferation. (**A**) shRNA-mediated knockdown of TCF7L1 reduces protein expression within 24 hours of induction by addition of doxycycline (Dox). (**B**) CRISPR/Cas9 was used to completely eliminate TCF7L1 protein expression, without inhibiting expression of other TCF/LEF factors (Supplementary Fig. S2). (**C**) TCF7L1 knockdown significantly slowed the growth of HCT116 cells within 48 hours of shRNA induction. (**D**) HCT116 cells with reduced (sh-TCF7L1) or eliminated (TCF7L1-Null) expression of TCF7L1 had significantly reduced colony growth (*P < 0.05, **P < 0.01, compared to Control). These cells formed fewer colonies with a smaller average colony size. Representative images are shown below quantification. Average and standard deviation were calculated across three independent experiments with at least three replicates each. (**E**) Loss of TCF7L1 affected cell cycle progression, causing a significant increase in G0/G1-phase cells, with a concomitant reduction in S-phase. (*P < 0.05; **P < 0.01 compared to HCT116 control for the respective cell cycle phase.) (**F**) Representative cell cycle images generated with ModFit.

Having confirmed that TCF7L1 represses Wnt signaling in HCT116 cells, we utilized two approaches to determine whether its loss had any effect on the cells. First, we generated cell lines expressing a doxycycline-inducible shRNA targeting TCF7L1^23^, which rapidly knocked down TCF7L1 mRNA (Supplementary Fig. S2) and protein (Fig. 2A) within 24 hours of induction. In addition to TCF7L1 knockdown, we also used CRISPR/Cas9 gene editing technology^24^ to generate cell lines completely lacking functional TCF7L1 protein (“TCF7L1-Null”) (Fig. 2B) without targeting other TCF/LEF transcription factors (Supplementary Fig. S2). Screening of clonal lines that were successfully transfected with the Cas9 vector revealed a loss or reduction of TCF7L1 protein expression in multiple clones, giving us increased confidence that the TCF7L1 gene was successfully targeted by this strategy. (Supplementary Fig. S2).

Knockdown of TCF7L1 significantly reduced HCT116 growth; the cell doubling time slowed from 24.8 hours to 31 hours, and four days after addition of doxycycline, there were 40% fewer TCF7L1-knockdown cells than control cells (Fig. 2C). In addition, TCF7L1 knockdown or protein loss (TCF7L1-Null) impaired colony formation, as cells formed significantly fewer colonies with a smaller average colony size (Fig. 2D).

To determine why loss of TCF7L1 reduced CRC growth and colony formation, we checked whether apoptosis or cell cycle progression was being affected. Intriguingly, we found that loss of TCF7L1 did not promote apoptosis or necrosis, as there was no meaningful increase in Annexin-V (early apoptosis) or DAPI (late apoptosis or necrosis) by flow cytometry (Supplementary Fig. S2). Instead we found that cells with reduced TCF7L1 expression had altered cell cycle profiles, marked by a significant increase in G0/G1-phase cells (Fig. 2E,F). As Wnt signaling activity has been associated with differentiation in certain contexts^25^, we hypothesized that some of the genes activated in the absence of TCF7L1 were promoting a more differentiated phenotype, causing cells to stick in G0/G1-phase.

### TCF7L1-Null HCT116 cells have compromised xenograft tumor growth

**Figure 3:**
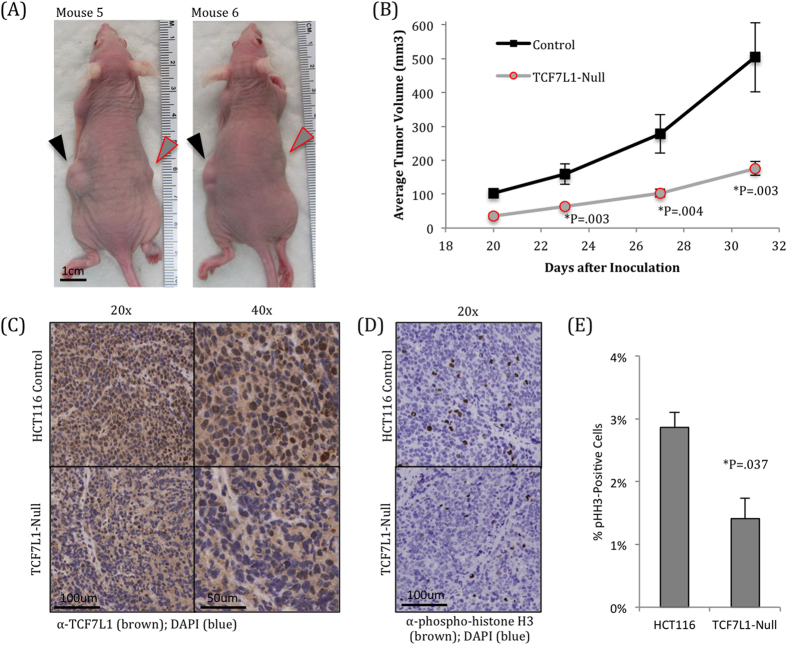
TCF7L1-Null cells have significantly reduced tumor growth. (**A**) 10,000 HCT116 control and TCF7L1-Null cells were inoculated subcutaneously into opposite flanks of mice and measured over 31 days once tumors became visible. Representative mice are shown 31 days after inoculation. Control tumors on the left flank (black arrowhead) are visibly larger than TCF7L1-Null tumors on the right flank (gray arrowhead with red border). (**B**) Quantification of xenograft tumor sizes shows that TCF7L1-Null tumors were significantly smaller than control tumors. Data are tumor volume mean ± SEM, n = 25. (**C**) Immunohistochemistry for TCF7L1 shows visible nuclear staining in control tumors (top), which is largely absent in TCF7L1-Null tumors from the same mice (bottom). Shown at 20x (left) and 40x (right) magnifications. (**D**) Immunohistochemistry for phosphorylated histone H3, a marker of actively dividing cells, shows fewer dividing cells in TCF7L1-Null tumors. (**E**) Quantification of pHH3 staining, average and standard deviation were calculated from three tumor pairs.

Having observed these *in vitro* phenotypes, we next wanted to determine whether this phenomenon would translate into an *in vivo* xenograft model in mice. We injected 10,000 HCT116 control cells in the left flanks of 6–8 week old athymic nude female mice, and injected 10,000 HCT116 TCF7L1-Null cells into the right flanks of those same mice. Once tumors became visible, they were measured 2–3 times per week. We found a striking reduction in average tumor volume in the TCF7L1-Null tumors, culminating with a 65% reduction on day 31 (Fig. 3A,B).

Six mice with representative tumor pairs were selected, and their tumors were excised for further characterization. Immunohistochemistry for TCF7L1 showed a clear nuclear staining in control cells, which was absent in the null cells (Fig. 3C). We also stained tumor sections for phosphorylated histone H3 and found that TCF7L1-Null tumors had significantly fewer dividing cells (Fig. 3D,E).

We sequenced RNA isolated from these six tumor pairs, along with cultured HCT116 control and TCF7L1-Null cells (three biological replicates each). Principal component analysis revealed clear distinctions between both the HCT116 control vs. TCF7L1-Null cells, and the cultured cells vs. tumors, with strong similarity among replicates and more heterogeneity among tumor samples (Supplementary Fig. S3). We selected several genes that were significantly altered in the TCF7L1-Null tumors by RNA-Seq (Supplementary Table S2), and confirmed their expression by qPCR (Supplementary Fig. S3).

To better understand the function of genes that were differentially expressed in the TCF7L1-Null cells and tumors, we performed GO term and KEGG pathway analyses. Some of the biological processes affected in these cells included transcription, RNA metabolism, cell morphogenesis involved in differentiation, and several processes involving neuronal development and differentiation (Supplementary Fig. S3, Supplementary Table S3). While the inclusion of neuron processes may seem out of place, the Wnt pathway is a critical regulator of neuron differentiation^26^, and previous analysis of TCF target genes showed enrichment in genes involved in axon guidance^27^. Pathways affected in TCF7L1-cells include ECM-receptor interaction, adhesion, and genes involved in colorectal cancer. Several additional pathways identified, including calcium signaling, hypertrophic cardiomyopathy, and ARVC, are also enriched among TCF7L2 target genes in colorectal cancer^28^.

### Cancer stem cell marker expression is reduced in TCF7L1-Null cells

**Figure 4:**
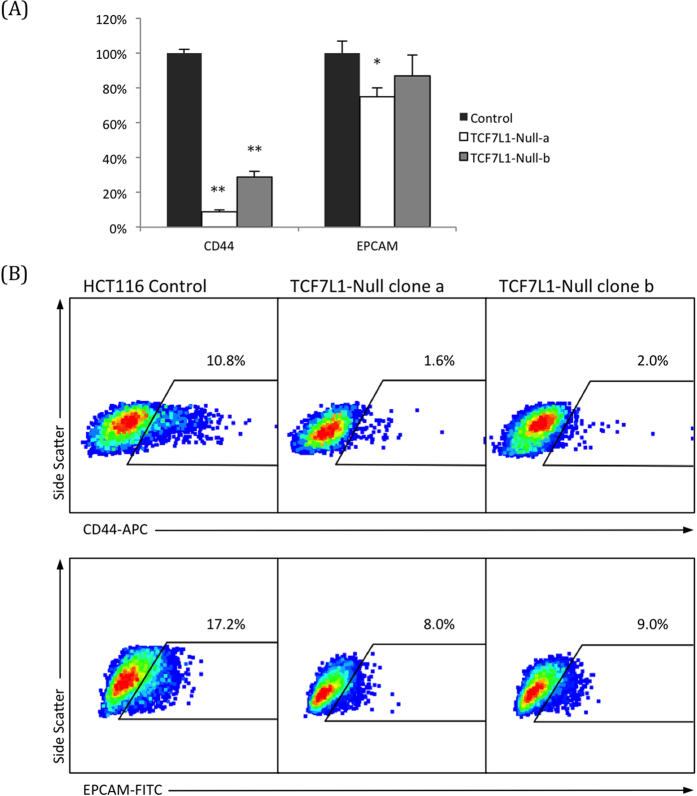
Expression of cancer stem cell markers is reduced in TCF7L1-Null cells. (**A**) qPCR confirmed that expression of CD44, a putative cancer stem cell (CSC) marker, was almost completely eliminated in TCF7L1-Null cells. Another CSC marker, EPCAM, had a modest reduction in TCF7L1-Null cells. (**B**) Flow cytometry with an APC-conjugated CD44 antibody revealed that TCF7L1-Null cells had significantly fewer CD44 + cells than control cells. (P < 0.01 for both clones, average was taken across three experiments.) FITC-conjugated EPCAM followed a similar trend, but with a less dramatic reduction in expression (P < 0.05 for both clones, average was taken across three experiments).

A top hit that was downregulated in TCF7L1-Null cells and tumors was CD44, a putative cancer stem cell (CSC) marker^29^ that was almost completely shut off in the absence of TCF7L1. The expression of a second CSC marker, EPCAM^29^, was also slightly downregulated. We performed qPCR for these genes in cultured HCT116 cells, using both TCF7L1-Null clonal lines, and found that while CD44 expression was nearly eliminated in both null clones, EPCAM was only slightly downregulated (Fig. 4A).

We performed flow cytometry for these CSC markers, and found that they were consistent with the qPCR findings. CD44 surface protein was largely absent in both null clones. EPCAM surface protein expression was also reduced in TCF7L1-Null cells, but the change was not as drastic as with CD44 (Fig. 4B). This suggests that while TCF7L1 has a clear function in suppressing pluripotency and self-renewal of embryonic stem cells^30^,^31^, this mechanism appears to be distinct from its regulation of CSC markers in CRC.

### Identification of candidate TCF7L1 target genes

**Figure 5:**
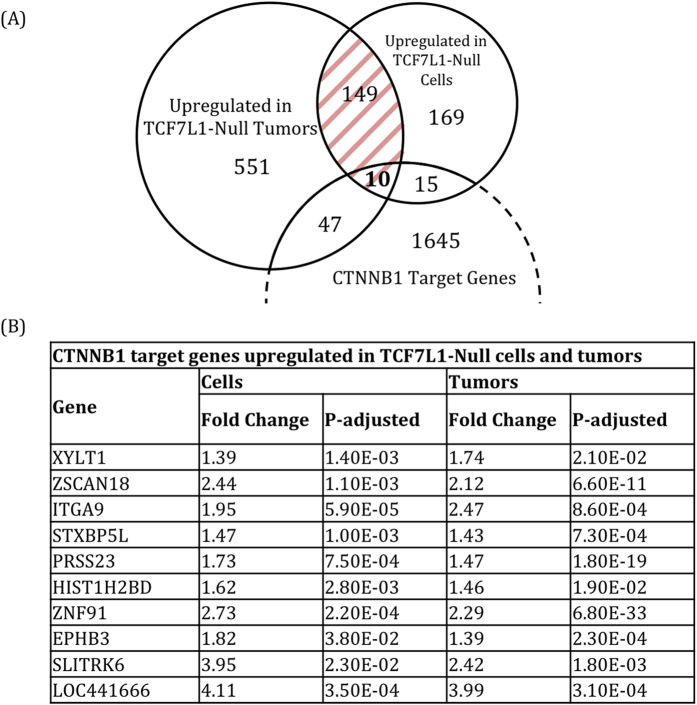
RNA-sequencing analysis reveals candidate TCF7L1 target genes. (**A**) Venn diagram showing genes significantly upregulated in TCF7L1-Null tumors and/or cells. The 757 genes upregulated in TCF7L1-Null tumors are shown in Supplementary Table S2. The 159 overlapping genes (shaded in red) are shown in the heat map in Supplementary Fig. S4. We identified ten genes from this group that have been previously described as direct targets of CTNNB1. (**B**) Table showing the ten CTNNB1 target genes that were significantly upregulated in TCF7L1-Null cells and tumors.

Having observed a reduction in HCT116 cell growth, colony formation, tumor growth, and CSC marker expression in the absence of TCF7L1, we searched for a mechanism to explain this phenotype. Since TCF7L1 is a transcriptional repressor, and we saw reduced growth in both TCF7L1-Null cells and tumors, we focused on the 159 genes that were significantly upregulated in both of these populations (Fig. 5A, Supplementary Fig. S4). To narrow our focus, we compared our data set to genes identified as direct targets of CTNNB1 in CRC cell lines^32^,^33^, of which TCF7L1 target genes should be a subset. This left us with 10 candidate TCF7L1 target genes that were significantly upregulated in both TCF7L1-Null cells and tumors, and have been identified as CTNNB1 target genes by ChIP-seq (Fig. 5B).

### Reduction of EPHB3 expression rescues growth of TCF7L1-Null cells

**Figure 6:**
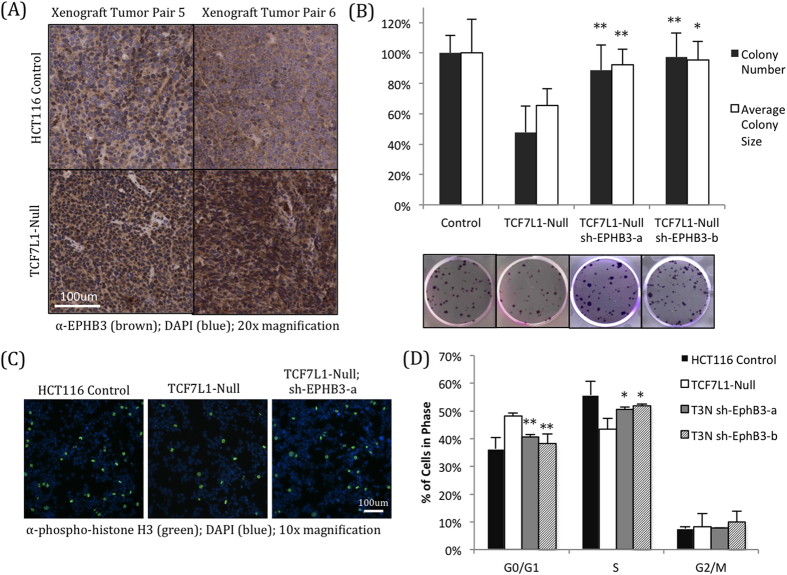
Knockdown of EPHB3 rescues growth of TCF7L1-Null cells. (**A**) Immunohistochemistry for EPHB3 shows elevated expression in TCF7L1-Null xenograft tumors (bottom) compared to control tumors from the same mice (top), reflecting the significant mRNA upregulation observed by RNA-sequencing and qPCR. (**B**) EPHB3 knockdown largely rescued colony formation of TCF7L1-Null cells. Colony number and average colony size was significantly higher than that of TCF7L1-Null cells, and almost returned to the levels of control cells (*P < 0.05, **P < 0.01, compared to TCF7L1-Null). Representative images are shown below quantification. Average and standard deviation were calculated across three independent experiments with at least three replicates each. (**C**) Immunofluorescence for phosphorylated histone H3 revealed that EPHB3 knockdown in TCF7L1-Null cells restores the percentage of actively divided cells near levels observed in control cells (quantified in Supplementary Fig. S6). (**D**) EPHB3 knockdown partially rescues the stalled cell cycle progression seen in TCF7L1-Null cells, with a significant reduction in G0/G1-phase cells and a corresponding increase in S-phase cells. (*P < 0.05, *P < 0.01, compared to TCF7L1-Null cells of respective cell cycle phase). Representative images shown in Supplementary Fig. S5.

In addition to its roles in promoting proliferation and stem cell self-renewal^3^, Wnt signaling can also promote differentiation in a variety of contexts, including in mouse embryonic stem cells^34^,^35^ and Paneth cells in the colonic and intestinal crypts^27^. Therefore, we were intrigued to see that cells and tumors lacking TCF7L1 had increased expression of EPHB3, a CTNNB1 target gene which has been shown to suppress tumor growth in mouse models of colorectal cancer^19^, reduce growth of cultured colorectal cancer cells^20^, and is enriched in lower grade areas of tumors^36^. Immunohistochemistry revealed stronger EPHB3 staining in HCT116 tumors lacking TCF7L1 (Fig. 6A), reflecting the increased mRNA expression observed by RNA-seq (Fig. 5B) and qPCR (Supplementary Fig. S3). In cultured HCT116 cells, we found that EPHB3 is expressed at the membrane and in the cytoplasm but not in the nucleus (Supplementary Fig. S5), fitting with its known role as a receptor tyrosine kinase^20^.

To determine whether EPHB3 activation was responsible for the reduced growth of TCF7L1-Null cells, we generated stable cell lines expressing shRNA constructs targeting EPHB3 in the TCF7L1-Null background. These cells had significantly reduced EPHB3 expression; in fact, mRNA and protein levels of EPHB3 were even lower than in the control HCT116 cells (Supplementary Fig. S5).

Functionally, EPHB3 knockdown was able to partially restore growth of TCF7L1-Null cells, as they formed colonies at a very similar rate and average size as control HCT116 cells (Fig. 6B). In addition, the number of dividing cells expressing phosphorylated histone H3 was similar to control cells (Fig. 6C, Supplementary Fig. S5), and normal cell cycle progression was also partially restored (Fig. 6D, Supplementary Fig. S5).

Notably, addition of Wnt3a-conditioned media did not affect expression of EPHB3, despite significant activation of the Wnt target gene AXIN2 (Supplementary Fig. S5). This supports our hypothesis that TCF7L1 represses a subset of Wnt target genes, including EPHB3, independent of CTNNB1 protein levels. Furthermore, it is consistent with our observations that TCF7L1 remains in the nucleus after Wnt3a addition, and helps to explain why Wnt3a addition does not cause the same growth-reduction phenotype as loss of TCF7L1.

Together, these findings indicate that activation of EPHB3 is at least partially responsible for reduced HCT116 colorectal cancer cell growth observed in the absence of TCF7L1. However, as the EPHB3 knockdown reduced expression below what was found in control cells, and the rescue was not complete, it is probable that other factors also contribute to this phenotype.

## Discussion

There is no dispute that Wnt pathway activation and CTNNB1 protein accumulation can be potent drivers of a number of cancer types, particularly colorectal cancer. However, given the large number of genes that are regulated by CTNNB1/TCF complexes, along with the numerous other proteins CTNNB1 interacts with, it is less clear how these functions promote or inhibit cancer progression in different contexts.

Our findings here highlight how in some contexts, excessive activation of specific CTNNB1 target genes can reduce CRC cell and tumor growth. Specifically, it raises the possibility that TCF7L1 may optimize CRC growth by limiting expression of tumor suppressor genes such as EPHB3. As this work was performed exclusively with HCT116 cells because of their expression of all TCF/LEF factors and their tractability, additional work will be required in order to determine whether these observations are relevant in other CRC cell lines and patient tumors.

In many ways, these findings reflect similar work done in breast cancer, where Slyper *et al*. found that TCF7L1 knockdown reduced cancer cell growth and that patients with high levels of TCF7L1 expression had poorer clinical outcomes^27^. Together, this work supports further investigation in the role of TCF7L1 in other cancer types, particularly those where Wnt signaling is known to regulate normal tissue homeostasis.

TCF7L1 may often get overlooked in CRC is because it is not significantly expressed in the small intestines of mice, one of the most common systems used to model CRC. However, TCF7L1 is expressed in the mouse colon; expression is concentrated in the base of the crypt where the stem cells are located, and decreases as cells migrate out of the crypt and differentiate^3^. TCF7L1 expression patterns in human colon crypts have not been investigated.

There is strong evidence that EPHB3 is directly regulated by TCF/LEF proteins. Schuijers *et al*.^32^ and Hatzis *et al*.^37^ both found by ChIP-Seq that TCF7L2 binds to a 5′ proximal enhancer 2–3 kb upstream of the transcription start site. In addition, Jagle *et al*.^38^ showed that this region is also occupied by markers of active enhancers (H3K27ac and p300), and their findings that EPHB3 is activated in early stage tumors but silenced in advanced tumors (corroborating work done by Battle *et al*.) is consistent with EPHB3 being both a target of Wnt signaling and a tumor suppressor. While the exact mechanism for tumor suppression is not understood, Cortina *et al*.^39^ showed that overexpression of EPHB3 and ephrin ligands in CRC cells caused them to compartmentalize, and that eprhin expression is critical to prevent tumor progression in mice with compromised APC function.

One major hurdle in understanding downstream Wnt signaling is the question of how different TCF/LEF factors bind different target genes, as the DNA binding domains are highly conserved. Recent evidence has shown that TCF7 and TCF7L2 can bind to DNA lacking consensus WREs through a C-clamp that is not present in LEF1 and TCF7L1^40^. In addition, ChIP-Seq studies identified a class of CTNNB1-binding transcriptional elements that can be disrupted by a dominant-negative TCF7L2, but do not strongly bind endogenous TCF7L2^32^. This suggests that there may be factors beyond the C-clamp that influence TCF/LEF binding to WREs and other DNA motifs.

Another challenge is in understanding what makes TCF/LEF factors function as transcriptional activators and repressors. LEF1 and TCF7 have been found to act primarily as activators; this can be explained by their low affinity for TLE/Grouch co-repressors^40^. On the other hand, TCF7L2 is often described as a transcriptional activator, but it has a strong affinity for TLE/Grouch proteins, and has also been found to repress Wnt pathway transcription^41^. While TCF7L1 is often viewed as a repressor, it has been shown to activate some genes in certain contexts^16^, and it is unclear why it has not been observed as an activator more frequently, given its strong similarity to TCF7L2.

Answering these questions is the key to understanding why CTNNB1/TCF activity can both promote and suppress tumor growth and stem cell-like properties, depending on the cellular and transcriptional context. As more academic labs and biopharma companies are investigating the use of Wnt pathway inhibitors to treat cancers, understanding these complex interactions can ensure that the best lead molecules are selected and used to treat appropriate patients, and improve therapy options for people diagnosed with colorectal cancer and other cancers with abnormal Wnt pathway activity.

## Materials and Methods

### Cell Lines and Medium

All cells were obtained from ATCC. HCT116 cells (ATCC CCL-247) were maintained in McCoy’s 5A Modified Medium; 293T cells (CRL-3216) were maintained in Dulbecco’s Modified Eagle’s Medium (“DMEM”); L-Wnt3a (CRL-2647) and L-Control (CCL-1.3) were used to make Wnt3a-conditioned medium, per ATCC protocol. All media was supplemented with 10% Tet System-Approved FBS (Clontech) and 1x penicillin-streptomycin. Cells were dissociated using TrypLE Express (Life Technologies) and were split at a ratio between 1:4 and 1:8 every 3–4 days.

### Statistical Analysis

Significance was determined using the Student’s two-tailed T test based on at least three independent experiments, unless otherwise noted. All error bars represent standard deviations, with the exception of xenograft tumor volume, which represent S.E.M.

### Transfection, Viral Packaging and Transduction

For TOPFlash assays, 1.25 × 10^4^ cells per well were reverse-transfected with 0.2 ul Lipofecatmine 2000 (ThermoFisher), 100 ng STF14x (Firefly Luciferase driven by 14 TCF/LEF binding motifs; a kind gift from Dr. Randy Moon, University of Washington, Seattle), 12.5 ng pRL-CMV (for normalization of cell number), and 5 nM siRNA (Ambion Silencer Select), in media lacking pen-strep. Cells were mixed with DNA, siRNA, and Lipofectamine in white 96-well plates, and spun down for 1 minute at 700 rpm. After 48 hours, Firefly and Renilla Luciferase were quantified using the Dual-Glo Luciferase Assay System (Promega) on a Perkin Elmer EnVision multimode plate reader.

For viral packaging, 5 × 10^6^ early passage 293T cells were transfected in a 10 cm dish with 20 ul Lipofecatmine 2000, 3 ug pCMV-delta8/9 and 300 ng pVSV-g with 3ug of viral plasmid, in 10 ml of DMEM supplemented with 20% FBS. After 24 hours, media was removed and replaced with 6 ml of fresh DMEM +20% FBS. After an additional 48 hours, this media was collected, spun down, and filtered through a 0.45 um filter.

For viral transduction, 2.0 × 10^5^ cells were plated in 6-well plates. 24 hours after plating, media was removed and replaced with 1 ml of fresh media containing 2 ug/ml polybrene. 500 ul of filtered viral media was added to the cells, and plates were spun down for 1 minute at 700 rpm. 24 hours later, the viral media was removed, cells were allowed to recover for one day, then were selected for at least 48 hours with media containing 2 ug/ml puromycin.

For inducible knockdown cell lines (sh-CTNNB1 and sh-TCF7L1), we cloned shRNA sequences identified by the Broad Institute RNAi Consortium (Supplementary Table S1) into the Tet-pLKO-Puro plasmid, a gift from Dmitri Wiederschain (Addgene plasmid #21915). Stable knockdown plasmids (sh-EPHB3) were obtained from Sigma (TRCN0000199452 and TRCN0000011020, in a pLKO.1 backbone).

### CRISPR/Cas9-Mediated Knockout

For generating TCF7L1-Null cells, a 20-bp guide sequence targeting the first exon of TCF7L1 was identified using the Zhang Laboratory MIT CRISPR Design Tool. Complementary synthetic oligonucleotides with 5′ overhangs (Supplementary Table S1) were annealed and ligated into the pSpCas9(bb)-2A-GFP (PX458) vector, a gift from Feng Zhang (Addgene plasmis # 48138), as described by Cong *et al*.^42^. The ligation mixture was transformed into One Shot Stbl3 cells, plasmid DNA was extracted from the clonal colony, and the sequence was verified. 100,000 HCT116 cells were reverse transfected in 6-well plates with 1 μg plasmid DNA using Lipofectamine 2000. After 48 hours, cells were sorted for GFP expression using a BD MoFlo XDP into 96-well plates, one cell per well, and clones were expanded and screened for absence of TCF7L1 protein by western blot analysis to isolate a clonal TCF7L1-Null cell line. The region surrounding the guide sequence in Exon 1 was amplified by PCT and sequenced. Sequencing was performed by Eton Bioscience.

### Colony Formation Assay

Cells were plated in 6-well plates at a density of 250 cells per well. Media was changed every 48 hours. Eight days after plating, cells were fixed with 1% glutaraldehyde, washed with PBS, and stained with 0.5% Crystal Violet. Plates were imaged on a Nikon TE2000-E microscope with a Coolsnap HQ2 camera. Automated quantitative image analysis was performed using the JOBS module (NIS Elements; Nikon).

### Western Blotting

Whole cell lysates were collected using RIPA buffer with protease and phosphatase inhibitors. Subcellular fractions were collected per manufacturer’s instructions using the Pierce Subcellular Fractionation Kit for Cultured Cells. Protein concentrations were determined using the Pierce BCA Protein Assay Kit. Identical protein quantities were loaded onto precast 4–20% Tris glycine gradient gels (BioRad) for electrophoresis. Gels were transferred onto nitrocellulose membranes and incubated overnight at 4C with one or more of the following antibodies: mouse anti-β-catenin (Sigma 15B8, 1:1,000), rabbit anti-TCF7L1 (Cell Signaling D15G11, 1:200), rabbit anti-LEF1 (Cell Signaling C12A5, 1:200), rabbit anti-TCF7 (Cell Signaling C63D9, 1:200), rabbit anti-TCF7L2 (Cell Signaling C48H11, 1:200), rabbit anti-EPHB3 (abcam EPR8280, 1:200), mouse anti-tubulin (Sigma T9026, 1:500), rabbit anti-RNA Pol II (Santa Cruz N-20, 1:200), mouse anti-GAPDH (Santa Cruz 6C5, 1:500), rabbit anti-Na + /K + -ATPase α (Santa Cruz H-300, 1:200). Secondary detection was performed using IRDye infrared-conjugated antibodies, and blots were imaged using the Odyssey Infrared Imaging System (LI-COR).

### RNA Isolation, cDNA Synthesis, and qPCR

RNA isolation was performed using TRIzol reagent from cells cultured in 6-well plates, or from flash frozen tumor samples, which were ground into a powder in liquid nitrogen with a porcelain mortar and pestle. The aqueous phase containing RNA was collected and cleaned using an RNA cleanup kit (Qiagen) and was eluted in RNase-free water. cDNA was prepared using a high-capacity cDNA Reverse Transcription Kit (Applied Biosystems). qRT-PCR was performed per manufacturer’s instructions using Brilliant II SYBR green master mix with low Rox (Agilent) on an Mx3005p qPCR system (Agilent). Relative gene expression was normalized to readings for GAPDH for individual samples and analysis was performed as per the ΔΔCt method^43^. Primer sequences can be found in Supplementary Table S1.

### Xenograft Tumor Assay

6–8 week old athymic nude female mice were inoculated subcutaneously with 10,000 CRC cells – HCT116 control cells in the left flank, and TCF7L1-Null cells in the right flank. Once tumors became visible, tumor volume was measured every 2–4 days. This experiment was performed two separate times, with at least 10 mice in each experiment. Upon completion of the second experiment (day 31), animals were sacrificed, and six representative tumor pairs were removed and cut in half. One half of each tumor was fixed in 10% formalin for immunohistochemistry, and the other half was flash frozen in liquid nitrogen for RNA isolation.

All animal protocols were approved by the Memorial Sloan-Kettering Cancer Center Institutional Animal Care and Use Committee and the Research Animal Resource Center, and all procedures were performed in accordance with the approved guidelines at the MSKCC Antitumor Assessment Core.

### RNA Sequencing and Data Analysis

RNA was isolated from cultured cells and tumor samples, treated with DNaseI, and sequenced on an Illumina HiSeq 2500 at the NYULMC Genome Technology Center. For each sample, two technical replicates of paired-end reads were run in different lanes.

Quality control was performed on FASTQ files using FastQC^44^, and low quality sequences were removed using Trimmomatic^45^. Sequences were mapped to Ensembl’s GRCh38.p3 version of the human genome^46^ using Tophat2^47^ and Bowtie2^48^. BAM files were sorted and deduplicated using picard-tools^49^, and technical replicates were merged using samtools^50^.

R and Bioconductor^51^ were used to determine differentially expressed genes and plot heat maps. Gene count was calculated using Rsamtools^52^ and GenomicFeatures^53^. Lists of differentially expressed genes were determined using the nbinomTest in DESeq^54^, and were filtered for adjusted p-value < 0.05 and a log fold change cutoff >Log2(1.3) or <Log2(−1.3) to identify differentially expressed genes.

### Immunohistochemistry

Xenograft tumors were cut in half and fixed in 10% formalin immediately after being excised. Tumors were embedded in paraffin blocks, and 3um sections were prepared on slides. Slides were stained with TCF7L1 (abcam ab86175, 1:50) and EPHB3 (abcam 4A122D1, 1:1600), or phospho-Histone H3 (Sigma 369A-1). Quantification was performed using Leica Microsystems Tissue IA software.

### Flow Cytometry and Cell Cycle Analysis

For extracellular markers, 1.0 × 10^5^ cells in 100 ul were incubated in the dark on ice with CD44-APC (Miltenyi Biotec DB105, 1:50, one hour), EPCAM-FITC (BD Biosciences, 1:25, one hour) or Annexin-V-FITC (BD Biosciences, 15 minutes). Cells were washed again with PBS + 4% FBS, and resuspended (using 1x binding buffer for cells stained with Annexin-V) with 1ug/ml DAPI to stain dead cells (which were excluded from CD44-APC staining). Cells were analyzed using a BD LSRII (with UV laser).

For cell cycle analysis, 1.0 × 10^5^ dissociated cells were fixed in 500 ul of ice-cold 70% ethanol for one hour on ice. Cells were washed and permeabilized in PBS + 0.25% Triton-X for 15 minutes, stained with 20 ug/ml of Propidium Iodide with 10 ug/ml of RNase A for 30 minutes in the dark, analyzed using a BD FACSCalibur Pro, and quantified using ModFit LT software.

### Immunofluorescence

12,500 cells were plated in black 96-well plates with clear bottoms. After 48 hours, cells were fixed with 4% formaldehyde, permeabilized with PBS + 0.5% Triton-X, and stained with rabbit anti-phospho-Histone H3 (Ser10) (Cell Signaling, 1:200). Secondary detection was performed with Goat anti-Rabbit Alexa Fluor 488 antibody (1:1000) with DAPI (1:1000), and imaged on a Nikon TE2000-E microscope.

## Additional Information

**How to cite this article**: Murphy, M. *et al*. TCF7L1 Modulates Colorectal Cancer Growth by Inhibiting Expression of the Tumor-Suppressor Gene EPHB3. *Sci. Rep.* **6**, 28299; doi: 10.1038/srep28299 (2016).

## Supplementary Material

Supplementary Information

Supplementary Information
