# Supplementary material for: TCF7L1 Modulates Colorectal Cancer Growth by Inhibiting Expression of the Tumor-Suppressor Gene EPHB3
Source: Sci Rep. 2016 Jun 23;6:28299. doi: 10.1038/srep28299 (PMC4917863; doi:10.1038/srep28299)
Supplement: Supplementary Information [file srep28299-s1.pdf]

Supplementary Information

TCF7L1 Modulates Colorectal Cancer Growth by Inhibiting Expression of the Tumor-Suppressor Gene EPHB3

Matthew Murphy[1], Sujash S. Chatterjee[1], Sidharth Jain[2], Manpreet Katari[2], Ramanuj DasGupta[1,3]*

* *Correspondence should be addressed to: ramanuj.dasgupta@gis.a-star.edu.sg*

1. *New York University Langone Medical Center*
   Perlmutter Cancer Center
   Department of Stem Cell Biology
   522 1st Ave., Smilow Research Building, Rm 1206
   New York, NY, 10016, USA

2. *New York University*
   Department of Biology
   1009 Silver Center, 100 Washington Square East
   New York, NY, 10003, USA

3. *Genome Institute of Singapore*
   60 Biopolis Street
   Cancer Therapeutics and Stratified Oncology
   #02-01, Genome
   Singapore, 138672

Supplementary Table S1
List of DNA sequences used for qPCR, CRISPR/Cas-9, and shRNA knockdown.

Supplementary Table S2
List of the 757genes which were significantly induced in TCF7L1-Null tumors compared to control tumors. Average counts, fold changes, and significance are shown for each gene.

Supplementary Table S3
List of biological processes (GO term) and pathways (KEGG) significantly altered in TCF7L1-Null cells and tumors.

Supplementary Table S4
List of the 159 genes which were induced in both TCF7L1-Null cells and tumors compared to control cells. Average counts, fold changes, and significance are shown for each gene.

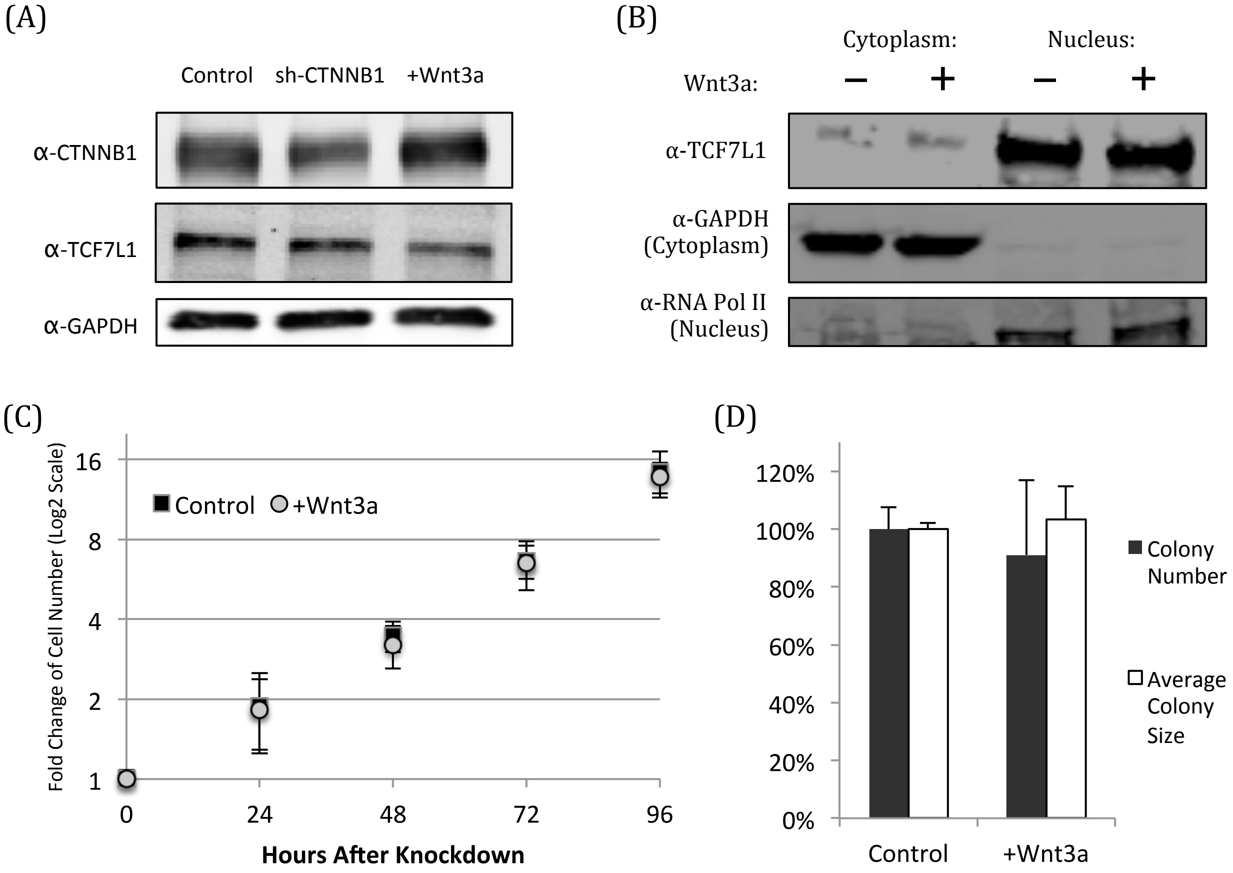

Supplementary Figure S1

(**A**) Induction of sh-CTNNB1 significantly reduces CTNNB1 protein within 48 hours (53% of control, P=.009) without significantly altering TCF7L1 protein (88%). Meanwhile, addition of Wnt3a-conditioned medium increased CTNNB1 protein levels (127% of control, P=.047), with a slight, but statistically insignificant decrease in TCF7L1 protein (80%, P=.34). Band intensity was normalized to GAPDH, and averages were calculated from four individual experiments. (**B**) TCF7L1 is localized to the nucleus in HCT116 cells, even in the presence of exogenous Wnt3a. (**C**) While Wnt3a activated Wnt pathway activity, the growth of cultured HCT116 cells was unchanged. (**D**) Wnt3a addition did not affect the number or average size of HCT116 colonies.

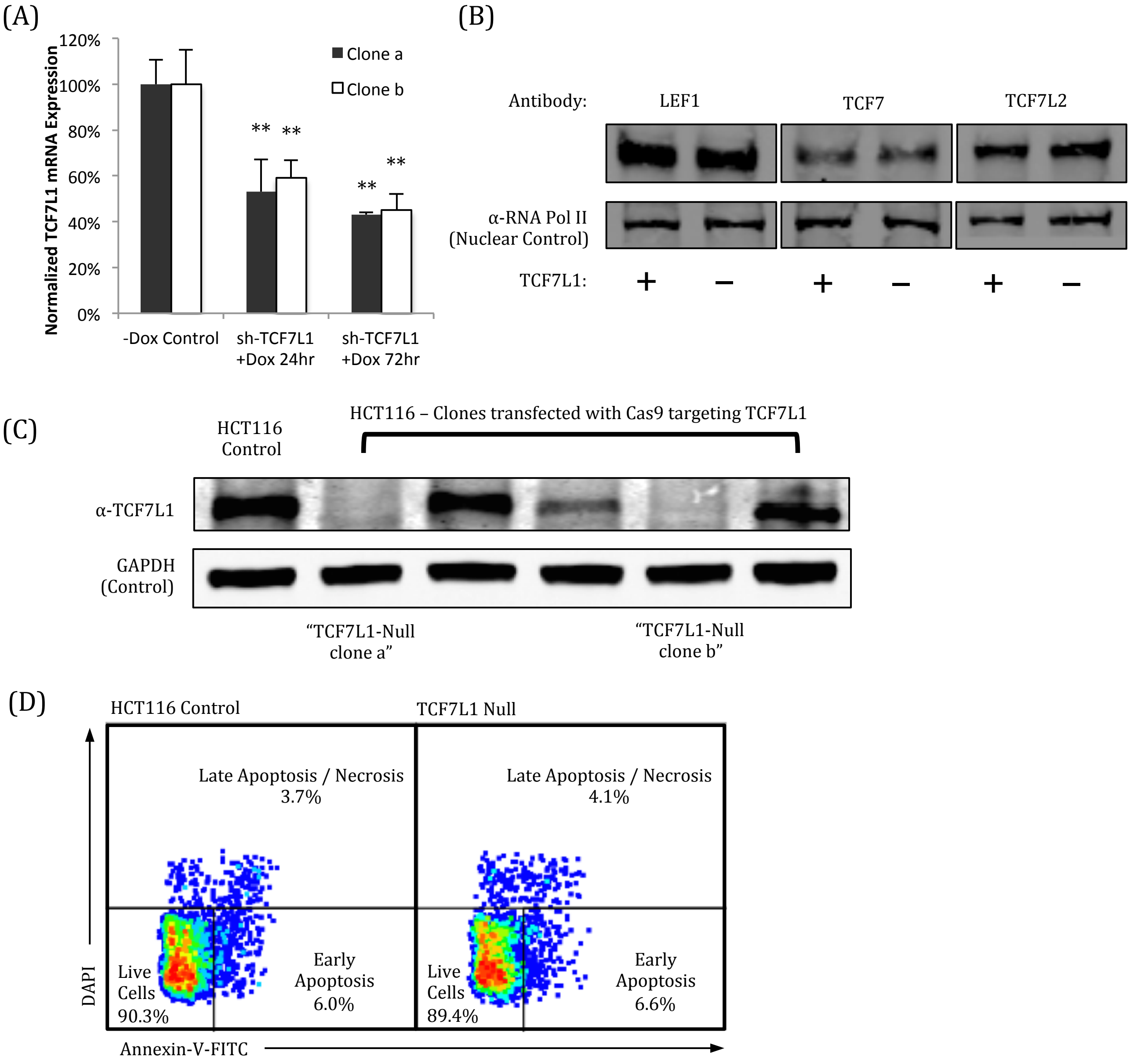

Supplementary Figure S2

(**A**) shRNA-mediated knockdown of TCF7L1 significantly reduces mRNA expression within 24 hours of doxycycline (Dox) addition (**P<.01). (**B**) CRISPR/Cas9-mediated loss of TCF7L1 protein did not reduce expression of other TCF/LEF factors. Nuclear fractions were used to concentrate TCF/LEF protein, and RNA Pol II was used as a nuclear loading control. (**C**) Clonal cell lines that were successfully transfected with the Cas9 vector targeting TCF7L1 were screened for TCF7L1 protein expression by western blot. We identified multiple clones completely lacking TCF7L1 protein, along with a probable heterozygous loss-of-function clone, giving us high confidence that our vector successfully targeted the TCF7L1 gene. TCF7L1-Null clone a was used for all subsequent studies unless otherwise noted. (**D**) Flow cytometry with Annexin-V-FITC (early apoptosis) and DAPI (late apoptosis / necrosis) revealed that loss of TCF7L1 did not induce cell death.

(A)

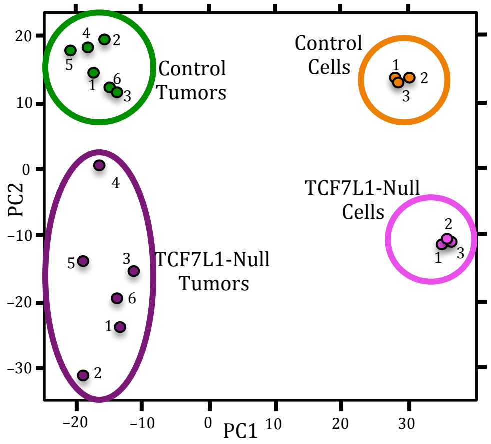

(B)

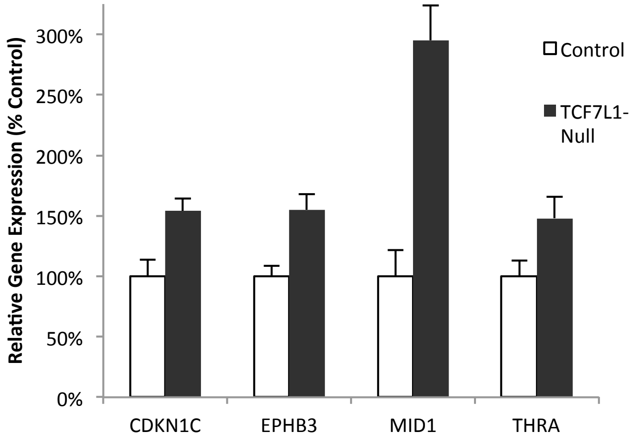

(C)

	GO term: Biological Processes	Count	P-Value
**Upregulated in TCF7L1-Null Cells**	regulation of transcription	81	6.90E-08
	regulation of transcription, DNA-dependent	62	1.00E-07
	regulation of RNA metabolic process	62	2.30E-07
	transcription	65	3.60E-06
	ossification	12	5.40E-06
	bone development	12	1.00E-05
	skeletal system development	18	4.70E-05
	neuron differentiation	21	9.00E-05
	biological adhesion	24	2.80E-03
	negative regulation of cell communication	12	4.40E-03
	**GO term: Biological Processes**	**Count**	**P-Value**
**Upregulated in TCF7L1-Null Tumors**	regulation of transcription, DNA-dependent	92	2.10E-05
	regulation of RNA metabolic process	92	4.80E-05
	metal ion transport	33	1.10E-04
	neuron differentiation	31	1.90E-04
	response to virus	13	3.00E-04
	transmission of nerve impulse	26	3.50E-04
	cell morphogenesis involved in differentiation	20	6.20E-04
	calcium ion homeostasis	17	6.30E-04
	cell morphogenesis involved in neuron differentiation	18	7.30E-04
	calcium ion transport	14	1.00E-03
	**KEGG Pathway:**	**Count**	**P-Value**
**Upregulated in TCF7L1-Null Cells OR Tumors**	ECM-receptor interaction	13	3.80E-05
	Dilated cardiomyopathy	13	9.60E-05
	Hypertrophic cardiomyopathy (HCM)	12	2.00E-04
	MAPK signaling pathway	21	1.30E-03
	Focal adhesion	17	2.20E-03
	RIG-I-like receptor signaling pathway	9	3.70E-03
	TGF-beta signaling pathway	10	3.80E-03
	Arrhythmogenic right ventricular cardiomyopathy (ARVC)	9	5.60E-03
	Intestinal immune network for IgA production	7	7.70E-03
	Calcium signaling pathway	13	2.40E-02
	Colorectal cancer	8	3.10E-02
	Cell adhesion molecules (CAMs)	10	4.70E-02

Supplementary Figure S3

(**A**) PCA plot showing clustering of sequenced control and TCF7L1-Null cell and tumor samples. (**B**) qPCR confirmed expression changes of selected genes found to be upregulated in TCF7L1-Null tumors by RNA-seq. cDNA from the six tumor samples for both conditions were pooled together. (**C**) Top hits from GO term and KEGG pathway analysis of genes upregulated in TCF7L1-Null cells and/or tumors. For GO terms, top 10 biological processes with at least 10 hits are shown. For KEGG pathways, all significant hits are shown.

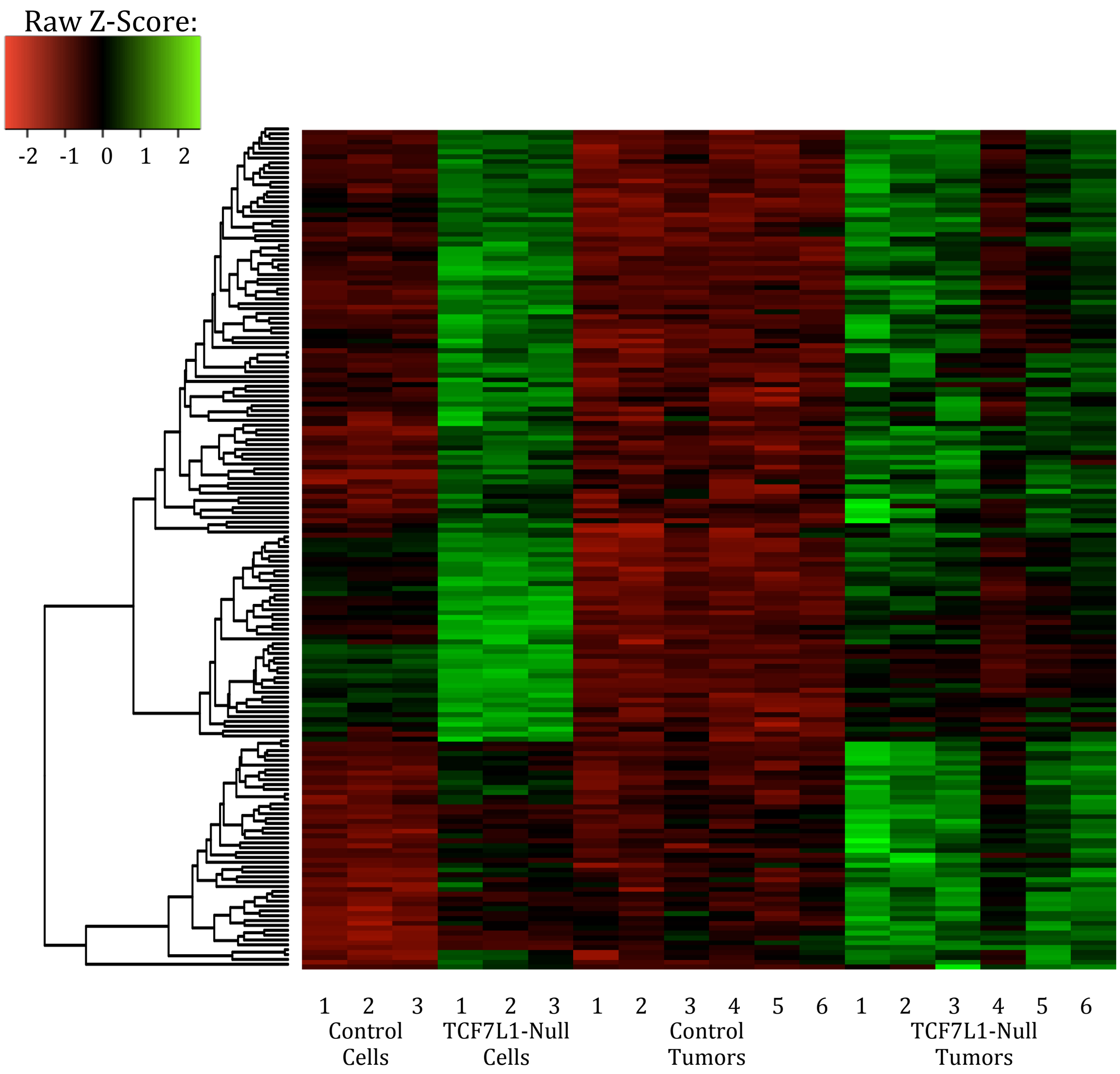

Supplementary Figure S4

Heat map showing the 159 genes that were significantly upregulated in both TCF7L1-Null cells and tumors, compared to their respective controls. Data for these genes can be found in Supplementary Table S4. As seen by PCA plot (Supplementary Fig. 3), TCF7L1-Null Tumor 4 had a poor correlation to other TCF7L1-Null tumor samples, but other replicates were highly similar.

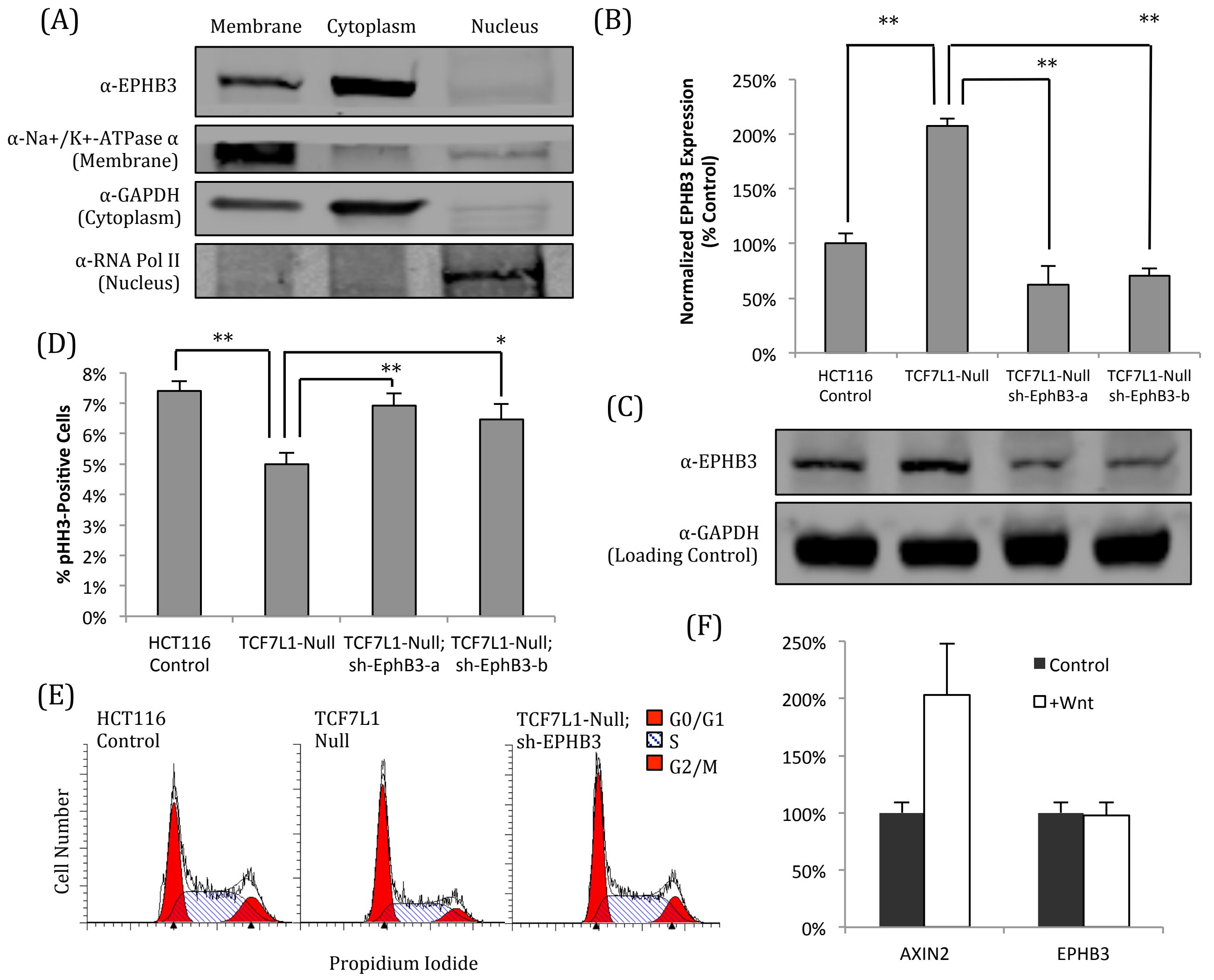

Supplementary Figure S5

(**A**) Western blot for EPHB3 in subcellular fractions of HCT116 control cells shows that the protein is found both at the membrane and in the cytoplasm, but not in the nucleus. (**B**) qPCR shows that cultured TCF7L1 cells have significantly higher EPHB3 expression than control cells (**P<.01), and shRNA-mediated knockdown with either of two shRNA clones significantly reduces mRNA levels (**P<.01). Notably, EPHB3 levels are reduced even below the levels seen in control cells. (**C**) Western blot for EPHB3 confirmed the trends seen above by qPCR. GAPDH was used as a loading control. (**D**) Quantification of phospho-histone H3 immunofluorescence in Figure 4C confirms a significant reduction of dividing cells in TCF7L1-Null cells, which is rescued after knockdown of EPHB3 (*P<.05, **P<.01, average of three experiments). (**E**) EPHB3 knockdown in TCF7L1-Null cells restores a cell cycle profile similar to control cells. Representative cell cycle images (quantified in Fig. 6D) generated with ModFit using flow cytometry data for DNA content using propidium iodide. (**F**) qPCR shows that while the Wnt target AXIN2 is upregulated 48 hours after addition of Wnt3a-conditioned medium, EPHB3 remains unchanged.
